# Anti-Interference Deep Visual Identification Method for Fault Localization of Transformer Using a Winding Model

**DOI:** 10.3390/s19194153

**Published:** 2019-09-25

**Authors:** Jiajun Duan, Yigang He, Xiaoxin Wu, Hui Zhang, Wenjie Wu

**Affiliations:** School of electrical engineering and automation, Wuhan University, Wuhan 430072, China

**Keywords:** Convolutional Neural Network (CNN), MobileNet-V2, fault localization, interference, Frequency Response Analysis (FRA), diagnosis, power transformer

## Abstract

The idea of Ubiquitous Power Internet of Things (UPIoTs) accelerates the development of intelligent monitoring and diagnostic technologies. In this paper, a diagnostic method suitable for power equipment in an interference environment was proposed based on the deep Convolutional Neural Network (CNN): MobileNet-V2 and Digital Image Processing (DIP) methods to conduct fault identification process: including fault type classification and fault localization. A data visualization theory was put forward in this paper, which was applied in frequency response (FR) curves of transformer to obtain dataset. After the image augmentation process, the dataset was input into the deep CNN: MobileNet-V2 for training procedures. Then a spatial-probabilistic mapping relationship was established based on traditional Frequency Response Analysis (FRA) fault diagnostic method. Each image in the dataset was compared with the fingerprint values to get traditional diagnosing results. Next, the anti-interference abilities of the proposed CNN-DIP method were compared with that of the traditional one while the magnitude of the interference gradually increased. Finally, the fault tolerance of the proposed method was verified by further analyzing the deviations between the wrong diagnosing results with the corresponding actual labels. Experimental results showed that the proposed deep visual identification (CNN-DIP) method has a higher diagnosing accuracy, a stronger anti-interference ability and a better fault tolerance.

## 1. Introduction

With the advancement of intelligent power equipment and Ubiquitous Power Internet of Things (UPIoT), it is urgent to study an accurate, intelligent, and adaptive fault diagnosing method suitable for the interference environment [1]. The power transformer is an indispensable and vital part in a power system, meanwhile, it is so fragile that numerous factors may lead to breakdown [2,3]. Research on its health management is very important [4]. 

Previous studies proposed many useful transformer condition assessment techniques [5], among which, Frequency Response Analysis (FRA) has received a great attention since there are many advantages: It is a non-intrusive test and very sensitive, even slight, deformations could be displayed on the response curves [6]. Research of transformer FRA diagnosing methods in recent years has mainly focused on how to find a reliable algorithm to interpret the transformer status [7] by extracting frequency response (FR) features of different kinds of faults [8,9]. Research on FRA compared in [10] were divided into two categories: the first aspect focuses on transformer model simulations [7,11], including research on 2D [12] and 3D models [13] or equivalent circuit for transformers [14]. Works in [15] proposed an artificial bee colony search algorithm to accurately represent the circuit parameters of transformer windings. In [16], data acquired from measurements to deduce the design parameters of single-phase distribution transformers was used, and then [17] further study of the parameter estimation methods to achieve higher accuracies was done; and the second aspect is the researches on diagnosing algorithms dealing with measured response data [18], which includes various statistical indicators such as cross-correlation coefficient [19], extreme points [20], etc. For example, [20] used M-sequence to describe the coefficients, poles, and zeros of transfer function, which were used for diagnosis. These methods belong to the traditional research for FRA. They can obtain transformer winding models and testing results, which provide theoretical supports for exploring the law of faults. Traditional diagnosing methods could be helpful, but they are not intelligent enough and less adaptable during the diagnosing procedures, because their criteria are limited to a certain transformer and call for experienced personnel [21]. 

Researchers are also discovering more intelligent methods. Lots of trained algorithms were adopted to identify faults in transformers, such as Probabilistic Neural Network (PNN) [22], Artificial Neural Network [23] and Digital Image Processing (DIP) methods [24,25,26]. Trained networks have shown effects during fault diagnosing processes, but their feature extraction abilities are still not good enough to achieve high accuracies and can only address one specific issue [27]. Digital image processing (DIP) methods could help standardize, automate, and enhance the fault interpretation process [25]. In recent years, it has gradually attracted the attention of researchers. At present, researchers who considered FRA diagnosing theories based on graphical representations mainly include: Abu-Siada Ahmed [24,28,29], Zhongyong Zhao [21,30,31], and Aljohani Omar, [25,26,32]. Research in [30] combined a graph theory method (binary morphology) and extreme point extractions to obtain diagnostic features of FRA. Graph theory methods are effective to cope with massive data [7]: image processing methods adopted in [33] were used to locate transformer radial deformations, and in [26] DIP were adopted to combine the magnitude with phase plots of the measured FRA signature into one polar plot. Therefore, more features could be extracted, and the diagnosing results could be more accurate. These DIP methods are relatively effective but the adaptabilities of these computation processes are still not good. They could be considered as a combination of traditional diagnostic methods + DIP. Researchers have not combined intelligent methods with DIP yet. Although some researchers used shallow neural networks or optimization algorithms in Artificial Intelligence (AI) to diagnose FR curves, they still needed to establish a set of rules for a specific dataset to find the regular patterns. This procedure requires complicate tests and analyses of the devices to be diagnosed. 

In general, current diagnostic methods still have the following problems:

(1) Existing diagnostic methods (traditional/AI or traditional + DIP) have poor applicability, they are difficult to form uniform rules. They need to design diagnostic rules and methods according to the equipment to be diagnosed [34]. Latest researches focused on the optimization of diagnostic rules through intelligent algorithms [35]. But researchers have rarely combined intelligent methods with DIP yet. Besides, even the intelligent methods are not adaptable.
(2) The background interferences are not taken into considerations. For offline FRA, after obtaining the response curves, they need to be transmitted to an equipment with fault diagnostic algorithms installed whether it is based on traditional methods, intelligent algorithms or the proposed deep learning DIP processes. In addition, with the advancement of UPIoT, there have been studies on online FRA in recent years [14]. In that case, the obtained data needs to be sent directly to the cloud computation platforms for fault diagnosis. Whether in the case of offline or online, the data set needs to be transmitted wirelessly or by wires before the diagnosis process. Therefore, Researches on fault diagnosis of power equipment need to take background noises into account [36,37]. Harsh interference would impose a significant impact on the quality and reliability of data [38]. In recent years, Research on improving anti-interference performance has made some progress [39]: for example, the noise reduction method [40], calculation of interference intensities [41,42] and noise reduction algorithms, such as the adaptive stochastic resonance filter [43] and Hilbert time-time (IHTT) transformations [44]. But they are unavailable for transformer FRA procedures because there exist only slight differences between the FR curves. Fault characteristics would be overwhelmed during the de-noising processes. Besides, power equipment such as transformers are in a high voltage, strong magnetic field environment, which tend to generate relatively large environmental white noises. For these reasons, traditional FRA diagnostic methods are difficult to put into applications: it is easy for the traditional diagnostic methods to be submerged under noise and go out of order.
(3) Although there are lots of guidelines and articles to implement FRA, only in recent years a few of them studied its localization methods [45,46]. It is necessary to provide positioning information of the faults for further intelligent diagnosing systems. An effective fault localization method is by comparing the tested FRA signatures of different winding sections with the fingerprint curves: larger variation (longer distance) means that there is lower possibility for fault winding in this area [46,47]. This idea was also used in the localization studies of partial discharge [48]. Works in [49] deduced the relationships between ladder network components and FR function, obtaining the features at different nodes, so that fault location can be acquired. And there’s another type of FRA localization method: by deliberately setting internal faults and then investigating the influence of various fault locations to the FR curves [50]. The studies are belonging to the traditional + localization methods, however, which require specific rules or inferences for different transformers [51]. Fault localization via graphical method could standardize and visualize diagnostic process, and reduce the interferences [52], which aroused attentions in recent two years. The concept ‘fault identification’ includes both fault type classification and fault localization. The current localization researches are based on statistical indicators, which are not intelligent enough and have poor adaptability [53].

In addition, the fault diagnostic information has not been made full use in existing researches. When the diagnosing result does not equal to the actual condition, it is necessary to excavate the useful information. 

In order to solve the problems (1)–(3) above, this paper proposed an intelligent fault diagnosing and localization method for transformer FRA based on deep learning + DIP. A data visualization theory and a deep learning method based on MobileNet-V2 were introduced to deal with fault identification problems: including fault type classification and fault localization. Compared with previous researches, proposed CNN-DIP method could standardize and facilitate the diagnosing process while achieving higher accuracies because the deep CNN could extract fine features automatically. Furthermore, this paper compared the anti-interference abilities of the proposed CNN-DIP method with the traditional method. Deeper information of diagnosing results was analyzed so that even the wrong diagnosing results could provide valuable information. 

The rest of this paper are organized as follows: Section 2 introduced the proposed deep visual identification (CNN-DIP) method and basic concepts. Section 3 conducted the experiments and analyses to compare different methods. Finally, conclusions were given in Section 4. 

## 2. Deep Visual Identification Method 

**Figure 1:**
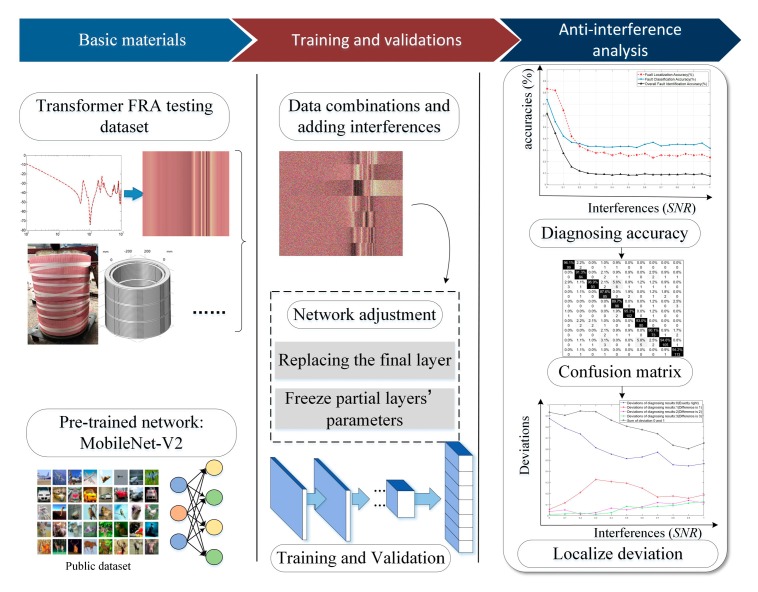
Graphical Abstract. The proposed deep visual identification (CNN-DIP) method includes the first two steps. And the third step conducts the anti-interference and fault tolerance analysis. FRA = Frequency Response Analysis; SNR = Signal-to-Noise Ratio.

The structure of the proposed deep visual identification (CNN-DIP) method is shown in Figure 1. 

### 2.1. Acquisition of Transformer FRA Graphical Dataset

The transformer of this paper was based on a customized winding, which is convenient for FRA and other fault diagnostic researches. It has 36 cakes, with 10 turns of continuous windings for a cake. It is suitable for the transformer which capacity is 100 kVA, the rated voltage of high voltage side is 10 kV (current is 10 A), and the rated voltage of the low voltage side is 0.35 kV (current is 285.7 A). The winding parameters have been listed in detail in Table A1. 

**Figure 2:**
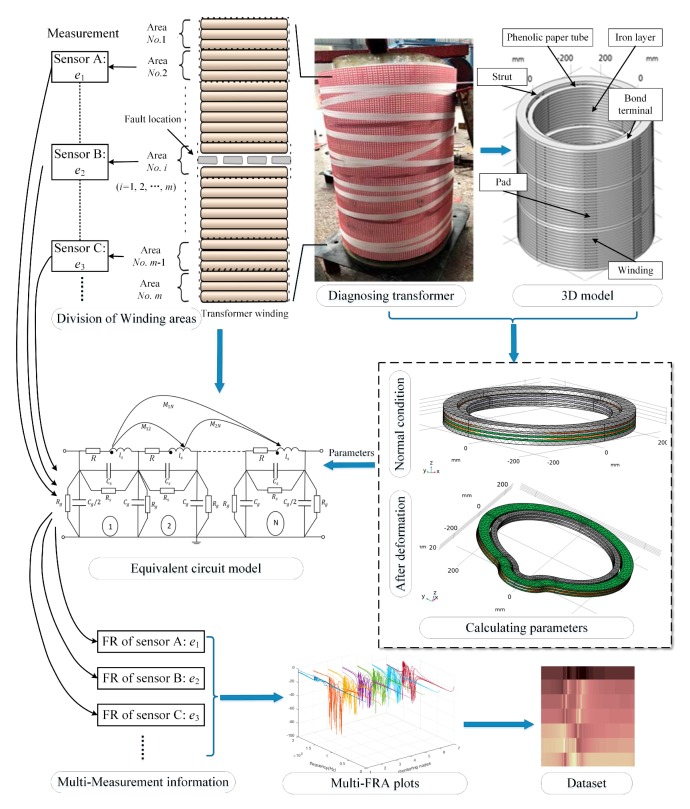
The dataset acquisition procedures, including winding area divisions, transformer modeling, parameter calculations of equivalent circuit; obtaining the frequency response (FR) curves and performing graphical representations.

The specific procedures for obtaining ‘transformer FRA testing dataset’ mentioned in the graphical abstract are shown in Figure 2. 

The structures and material characteristics of the windings must be considered in simulation model. And the winding disc is composed of flat Aluminum wire wrapped by insulation paper and the structural support of the winding is composed of stay and resilient pads. The structures of the winding and relative dielectric constants of the insulation materials are shown in Table A1. 

The multi-FRA image in Figure 2 was obtained by constructing an equivalent circuit model through MATLAB programs. First, according to the structural parameters of the transformer to be diagnosed, the 3D model in Figure 2 was established, and the winding parameters were calculated. Then the parameters were substituted into the equivalent circuit of transformer windings [12]. High-order mutual inductances are tiny, and they are usually omitted [19]. The training dataset was formed by combining the FR curves from different regions of the winding. At last, loops in programs were set to automatically get a large number of data sets. 

Finite element simulations were performed using the COMSOL Multiphysics simulation software. After the transformer winding model being established, the corresponding materials were set. Electrical parameters of the windings were calculated, including: the ground capacitance *C_g_*; the disc to disc capacitance *Cs* between the neighboring winding cakes; the self-inductance *L_s_* of each wire cake of the winding; the mutual inductance *M_i_*_(*i*+1)_ and *M_i_*_(*i*+2)_ between the cakes; the resistance *R* of each cake of the winding and *R_s_*, *R_g_* the resistances in parallel with the series and ground capacitances, respectively. When calculating parameters, assuming that no faults have occurred at first. Use 1 cake in the 3D model to calculate the resistance *R* and self-inductance *L_s_*. Use 2 cakes to calculate the mutual inductance *M_i_*_(*i*+1)_ and capacitance *Cs*. Use the iron core and a cake to calculate the capacitance to ground *C_g_*. Take 3 cakes to calculate the second order mutual inductance *M_i_*_(*i*+2)_ (such as *M*_13_). The values of insulation resistance of transformer windings (*R_s_*, or *R_g_*) are large (more than 1 MΩ) and nearly have no effects on FR characteristics. Therefore, they were set as a large value (*R_s_* = 15 MΩ or *R_g_* = 70 MΩ [12]) in the paper. 

The winding part (including all the entities, such as bond terminal, the pad, etc.) was divided into fine meshes during the 3D simulation, the smallest unit of which is 20 mm; the air part is automatically split. There exists a total of 48138 units with an average unit mass of 0.5267.

The major parameters obtained by solving the 3D models are shown in Table 1.

In order to obtain the FR curve, an integrated transformer winding circuit model considering mutual inductances and capacitances was established. Parameters of the same components in each winding section are equal to each other. The input voltage signal *U*_1_ of the port was set as the swept-frequency signal, and the output current signal of the other port was measured as *U_out_* so that the FR curve of the winding could be obtained, as shown in Equation (1):

(1) $$L=20\mathrm{lg}\frac{\left|U_{out}\left(f\right)\right|}{\left|U_{1}\left(f\right)\right|}=\left\{L\left(f\right)\right\}$$

where *L* is the transfer function, *f* is the testing frequency, *U*_1_ is the input voltage signal, and *U_out_* is the output voltage signal. If there is more than one detection point, the response curve of the detection point *k* is marked as *L_k_*, and the transfer functions acquired by all the detection points are combined into an FR matrix ***L***:

(2) $$L=\left[\begin{array}{c} L_{1}\\ \vdots \\ L_{k} \end{array}\right]$$

In this paper, the transformer shown in Figure 2 is evenly divided into seven detection areas, and the detection areas: *i* = 1, 2, … , N, (N = 7). The detection points are located at the end terminal of each area. 

### 2.2. Fault Localization Based on CNN or Space Relationship

We extracted the features of different FR curves in this section, while fault location varies. Because, under the harsh interference environment of transformers, subtle changes of FR curves may be submerged, it is necessary to carry out the study of a more profound and intelligent diagnostic method, which is less to be affected by the interferences. 

#### 2.2.1. Basic Theories of MobileNet-V2 and the CNN-DIP Method

**Figure 3:**
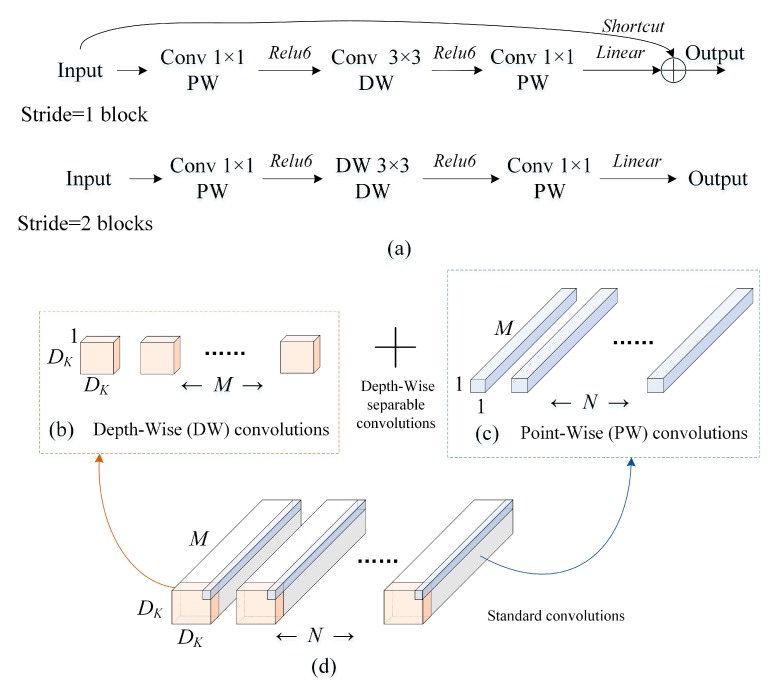
Basic blocks of the MobileNet-V2. (**a**) Basic blocks in MobileNet-V2; (**b**) Depth-wise Convolutional Filters; (**c**) Point-wise Convolutional Filters; and (**d**) Standard Convolutional Filters.

This section gives a brief idea about MobileNet-V2 architecture. The basic structure: ‘blocks’ of the network are shown in Figure 3a. 

The network architecture of MobileNet-V2 could operate under the condition without too much calculation processing resources with higher diagnosis accuracy [54]. The bottleneck layer of this network has an extension which includes the inverted residual connections [55]. This model extends the concept of Width Multipliers introduced in MobileNet-V1, which proposed Depth-wise Separable Convolutions [56]. Depth-Wise convolutions (in Figure 3b) and point wise convolutions (in Figure 3c) replaced the Standard convolution filters (Figure 3d). MobileNet-V2 mainly proposed two innovative improvements: 

Inverted residuals: An 1×1 convolutional layer served as ‘expanded’ layer is added before the Depth-Wise Convolution process, with the aim to increase the number of channels and to obtain more features; 

Linear bottlenecks: What is adopted finally is not layer *Relu*, but layer *Linear*, the aim of which is to prevent *Relu* from damaging features. This is because for negative input, output of *Relu* is zero; and its original features have been ‘compressed’, some of which would get ‘lost’ if repassing *Relu*.

Figure 3a are two types of connecting structure blocks of the network. Aiming at *stride* = 1 and *stride* = 2, the block structure varies slightly, mainly to match the dimensions of shortcut: when *stride* = 2, shortcut is not adopted.

Replacing the last layer of this network with learnable weights, a fully connected layer (FL) for this network), with a new FL with the same outputs number equal to the number of classes for diagnostic dataset. Then, the proposed CNN-DIP method in this paper only needs to input the training or validation dataset obtained according to Figure 2 into the network for training and the validation processes. 

#### 2.2.2. Spatial-probabilistic Mapping Relationship Based on Traditional Method

Traditional FRA fault diagnosis method is to compare the FRA test curves of the transformer to be diagnosed with the previous standard curve (the fingerprint) [57]. However, existing researches based on this method basically only judge whether there exists a fault. In this section, we improved the traditional method by comparing the peak features so as to obtain fault locations. 

For each monitoring node, its FR is defined as formula (1). Find the peaks of each monitoring node, i.e., the extreme values of each row in matrix ***L***. Define the feature vector ***L****_feature_* as:

(3) $$L_{feature}=\left\{{peak\left(L\right)\;|}_{keep\;extreme\;values}\right\}$$

Therefore, the response feature matrix was deduced as:

(4) $$L_{feature}=\left\{\begin{array}{c} L_{1\_feature}\\ \vdots \\ L_{k\_feature} \end{array}\right\}$$

The ***L****_fingerprint_* denoted the fingerprint results for each diagnosing point (in standard, normal conditions), and ***L****_k_feature_* represents the results of the *k*-th measurement point, which is on the other terminal of the winding. ***L****_i_feature_* and ***L****_i_fingerprint_* represent the information of the *i*-th point, respectively. The deviations between ***L****_feature_* and the fingerprint matrix ***L****_fingerprint_* were calculated to localize faults by a spatial-probabilistic mapping relationship. Therefore, the relevant fault localization probabilities are:

(5) $$P_{i}=\frac{1}{\sqrt[{\rho }]{\sum_{i=1}^{N}{\left|L_{k\_feature}-L_{k\_fingerpr\mathrm{int}}\right|}^{\rho }}}$$

where the parameter N represents the maximum number of features, which equals to the total number of areas. The *i*-th area of the winding was represented by *i*. Therefore, *P_i_* represents the relative possibilities indicating that the *i*-th node is on a fault condition. *ρ* is a variable parameter: when *ρ* = 1, formula (5) is based on the *Manhattan* distance, when *ρ* = 2, the *Euclidean* distance, and when *ρ*→*∞*, *Chebyshev* distance. These distances are defined as *Minkowski* Distance. According to simulation and comparisons, *ρ* = 2 achieved the best performance in this paper. 

The algorithm described in (5) directly assigned the differences to the support of faults. If there exist more than one measurement point, they are more sensitive and effective for nearer faults. A more accurate possibility allocating algorithm was proposed by introducing the impact index *δ*, shown in formula (6).

(6) $${P_{i\pm \delta }|}_{\delta =1,2,\cdots ,\Delta }=\frac{1}{1+\delta }\frac{1}{\sqrt[{\rho }]{\sum_{i=1}^{N}{\left|L_{i\_feature}-L_{i\_fingerpr\mathrm{int}}\right|}^{\rho }}}$$

where the impact index *δ* is determined by the deviations between the measurement and fingerprint data, and the maximum value Δ of the impact index *δ* is calculated by formula (7). This means that while the deviations increased, the fault possibilities on this area would be smaller, and the distribution of possibilities would be more scattered.

(7) $$\Delta =\mathrm{INT}\left(\sqrt[{\rho }]{\sum_{i=1}^{N}{\left|L_{i\_feature}-L_{i\_fingerpr\mathrm{int}}\right|}^{\rho }}\right)$$

where ‘INT( )’ represents the rounding operation. And if *δ* ≥ N, let *δ* = N.

Finally, the relative possibility index *P_i_* was normalized to obtain the fault support index *P_i_*^’^ through formula (8):

(8) $${P_{i}}^{\prime }=\frac{P_{i}}{\sum_{i=1}^{N}P_{i}}$$

### 2.3. Data Visualization Theory

Lots forms of dataset could be easily mapped into 2D or 3D coordinates and converted into images through simple transformations (gray images for 2D datasets and colorful images for 3D). The specific data visualization processes and their classifications are shown in Table 2.

**Figure 4:**
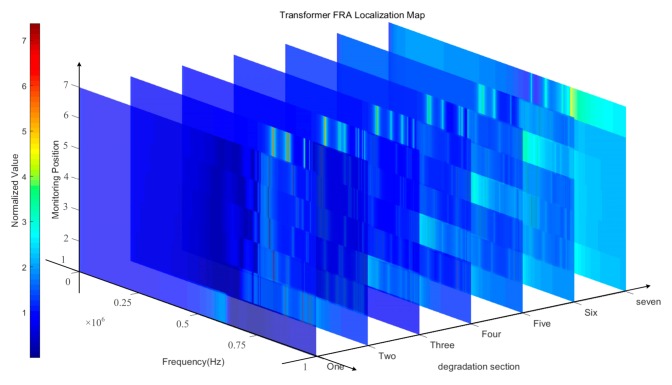
Feature map of transformer FRs while fault and monitoring locations vary, where the Z axis represents the normalized value, which is 20*log(the amplitude/the average value in normal state).

The monitoring information was processed and extracted by means of digital image processing (DIP). Denote the monitoring fault types as *j* and fault areas as *i*, then they were combined and written as ‘*ji*’, and the corresponding label is H*_γ_*. The label of the normal state is ‘00’, i.e., H*γ* = {00, 11, 12, ..., *ji*}, *γ* = 1, 2, ..., *l*, and *l* is the total number of labels. According to Table 2, the type of FRA data is ‘*waveform data*’. As the fault locations changed, the complete (while *k* = 1, 2, …, N) FR matrices ***L*** changed, as shown in Figure 4. 

It could be observed from Figure 4 that there is a positive correlation between the locations of measurement points and the fault winding sections. This phenomenon was clearly illustrated in Figure 4. Figure 4 combined four-dimensional information of FRA testing results: the magnitudes of resonances, frequencies, location of different monitoring nodes, and the location of different deterioration areas. The brighter part of each surface showed that there might exist a fault because the transfer functions near this frequency range is abnormal, and response signals here decay strongly faster than its surrounding areas. This significant characteristic can be utilized to extract deep features through the visual identification methods. 

## 3. Model Test and Results

Testing procedures in our work comprised three major parts. First, fault localization and diagnosing results based on deep visual identifications (CNN-DIP) or spatial probabilistic mapping relationships were obtained. And their diagnosing results were compared and analyzed while no interference existed. Then, as the noises continued to increase, the fault diagnosis and positioning effects of different methods were tested, and their capacities to resist interference were analyzed. Finally, fault tolerance of these methods was tested. 

In this paper, three fault types were set: the change of disc to disc capacitance *Cs*, the change of self-inductance *L_s_*, and the change of ground capacitance *C_g_*. It should be noted that these variations do not completely correspond to a certain type of fault. For example, the change in the disc to disc capacitance *Cs* between the cakes may be caused by a change in the distance between discs or by the winding deformations. In fact, the fault types could also be set as the gaps between cakes, the winding deformations, etc. 

### 3.1. Fault Identification Results without Considering Noises

#### 3.1.1. Traditional Spatial-Probabilistic Model

**Figure 5:**
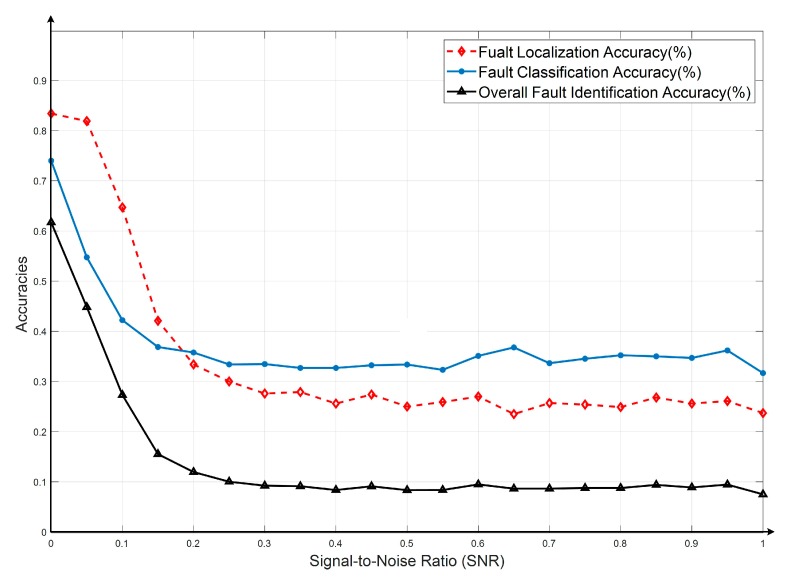
Fault identification results while SNR increases using traditional method.

The accuracies of the fault localizations were obtained via formula (6) and (8). First, in cases without interferences, 1000 times of random simulation tests in total were conducted. The fault types diagnosing accuracy was 74.0% and the accuracy of fault location is 83.4%. So, the total accuracy of fault recognition is 61.7%, as shown in the first column in Figure 5. 

#### 3.1.2. Deep Visual Identification via MobileNet-V2 

The FR curves were transformed into images, as shown in Figure 2. After the image augmentation process and the change of FL layers, they were input into the MobileNet-V2 for fault identification. Specific categories and quantities of the dataset were shown in Table 3. The samples (00) in the first row indicate the normal situations, and the remaining rows correspond to the three fault types for each fault location. The number of samples for different fault types is the same, so it is three times the first row. The dataset for fault identification based on MobileNet-V2 totally contained 5500 images each time (for each Signal-to-Noise Ratio (SNR)), among which 80% were for training and 20% for testing. 

**Figure 6:**
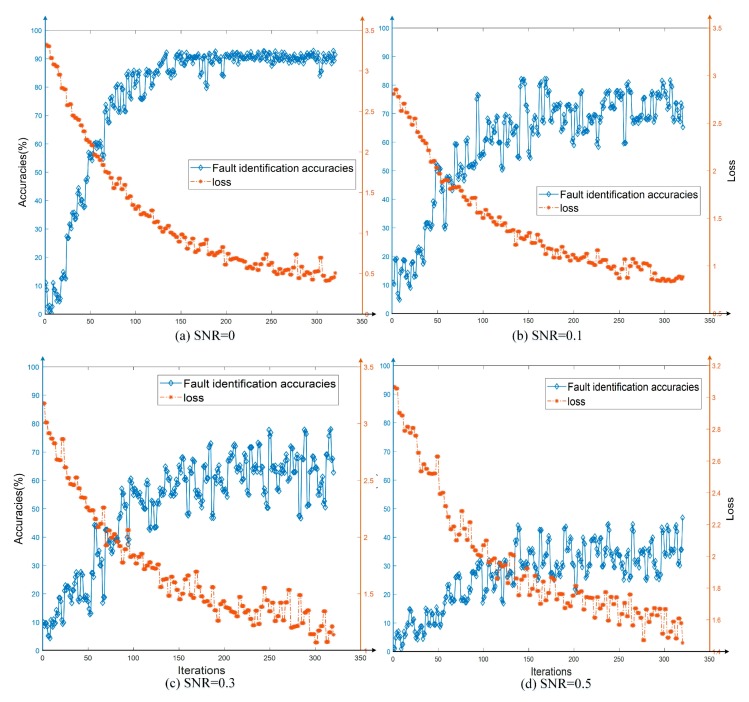
The validation procedures of the proposed deep visual identification (CNN-DIP) method (based on MobileNet-V2): (**a**) SNR = 0; (**b**) SNR = 0.1; (**c**) SNR = 0.3; and (**d**) SNR = 0.5.

The training and verification process of MobileNet-V2 model using the graphical dataset are shown in Figure 6a. 

It can be seen from Figure 6a that the fault identification accuracy reached 91.67%, and the network training process had a short rising period and could complete the training progress quickly. Since the transformer windings actually includes dozens or even hundreds of discs, it is difficult to achieve precise fault localization for each certain disc. Therefore, this paper studied the localization areas based on the division of regions. When there is a fault disc, the parameters of its area would be abnormal. If a more accurate localization result of the windings is necessary, the number of divided regions could be increased, and accordingly the training dataset must also be increased. 

Other training processes in Figure 6 are the cases after adding the interference, which would be discussed in detail in the next Section. 

### 3.2. Anti-Interference Analysis

In this section, the anti-interference abilities of transformer FRA diagnostic procedures based on traditional methods or deep visual identification methods (CNN-DIP) were considered. Their localization accuracies were tested and analyzed. Gauss White Noises were used in this paper to represent background interferences. 

Random noises characterize the additive interference signals generated by the strong magnetic field on monitoring environments. In this paper, in convenience of description, the amplitude ratio (signal-to-noise) is marked as the Signal-to-Noise Ratio: SNR. 

For the measurement response matrix $L_{feature}$ in formula (4), randomly additive noises with an average absolute value of SNR are included.

(9) $$L_{transfer}=L_{feature}+n\left(t\right)$$

where ***L****_transfer_* indicates the data transported from measurement sensors to the computer or a computation cloud, where fault diagnosis procedures are conducted. ***n***(*t*) is a matrix with the same size as ***L****_transfer_*. It contains the Gauss White Noises indicating the background interferences. This type of noise is added to the primitive monitoring information: $L_{feature}$. 

#### 3.2.1. Traditional Spatial-Probabilistic Model 

As the interference increasing continuously, the deviations of the fault diagnosing and localization accuracies by the traditional method are shown in Figure 5. It can be seen that with the interference increasing (increase of SNR), the diagnostic accuracies decreased sharply. When SNR raised to 0.2, the diagnosing result (11.9%) almost reached the minimum value. So, the traditional method has poor anti-interference abilities. 

#### 3.2.2. Proposed CNN-DIP Method 

**Figure 7:**
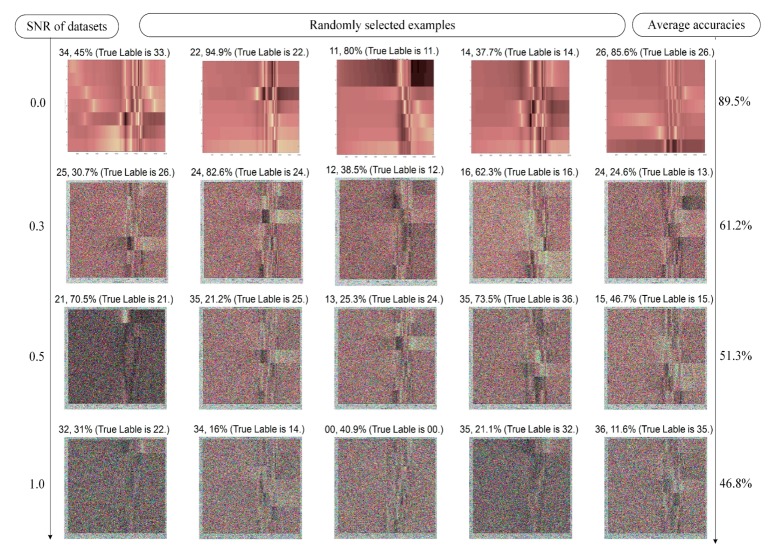
Randomly selected examples of the anti-interference abilities.

The fault identification accuracies (including fault-type diagnosis and fault location) when the interference increased (SNR increased) were calculated by the proposed CNN-DIP method. The training processes of various interferences are shown in Figure 6b–d, which represent the cases when SNR = 0.1, 0.3, and 0.5, respectively. For the convenience of observing, the fault identification accuracies and some results when SNR = 0, 0.3, 0.5, 0.7, and 1.0 were listed in Figure 7. Five identification results were randomly selected from the validation datasets for each SNR cases. It can be seen that when the noise intensity was increased to 50%, the features of the dataset to be diagnosed are already cannot be distinguished by the naked eye. 

It can be seen from the data in Figure 7 that the proposed CNN-DIP method based on deep visual features has stronger anti-interference abilities, which attenuates to the minimum value until the SNR reached 0.5. The proposed CNN-DIP fault identification method could simultaneously diagnose the fault types and the fault locations under strong noises. 

### 3.3. Deviation Distances of Diagnosis Error

In actual applications, the diagnostic results of fault locations would show a normal distribution with the mean value being the actual fault and the variance being as small as possible. Even when there are errors in the diagnostic results, it is still of reference value as long as the actual fault location is close to the diagnosed location. This indicates that the fault localization results have different fault tolerances. Therefore, in this section, the deviations of the localization results were compared via the two methods in this paper, without considering the fault-type diagnosing results.

#### 3.3.1. Traditional Method

**Figure 8:**
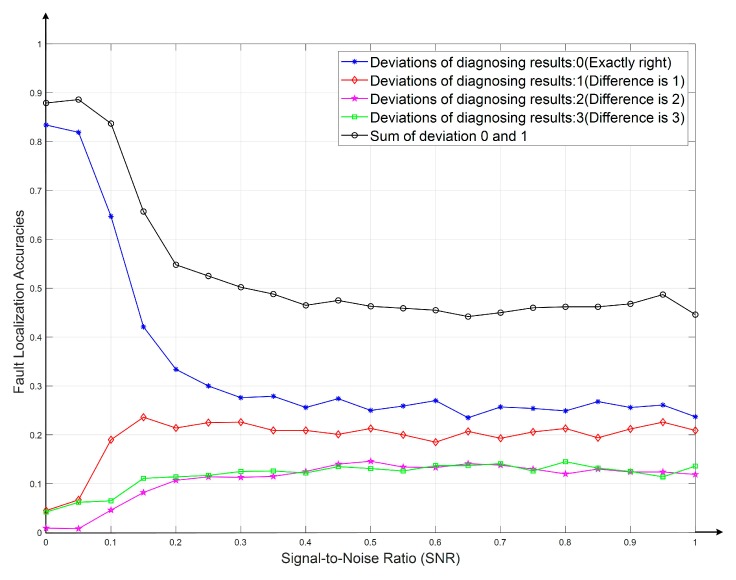
Deviations between actual fault sections and the localization results by traditional method.

For the traditional diagnostic method, the difference between the localization results of each tested sample and the correct locations was recorded. For example, if the diagnosed fault winding is No. 5 and the actual fault is in No. 3, the fault distance would be 2; while the diagnostic result is the same as the fault location, the fault distance is ‘0′. Deviations in cases of different noise strengths (SNR keeps increasing) were obtained by test, which were plotted in Figure 8. It could be seen from the figure that when the interference is very small, the wrong localization results are usually small, which means the localization results are more accurate. When the interference increases, the deviation increased sharply, and the dispersion of the localization results becomes larger, which means that the reference of the diagnostic results reduced significantly. 

#### 3.3.2. Proposed CNN-DIP Method

**Figure 9:**
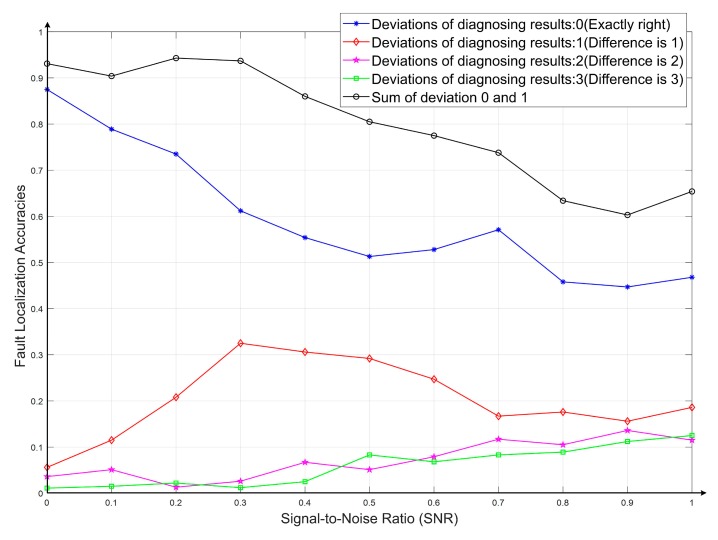
Deviations between actual fault locations and the diagnosed results.

The fault recognition results with CNN-DIP method based on MobileNet-V2 under different noises and interferences were calculated. The information of fault localization deviations was calculated by the confusion matrix of the diagnosed results for each level of noise. When testing the fault tolerances, the SNR was set to [0, 1] and the step size was set as 0.1. The final fault deviation curves are shown in Figure 9. 

It can be seen that when the interference (SNR) increased gradually, not only the accuracies dropped obviously slower than the traditional method, but the deviations of fault diagnosing labels also reduced significantly. This indicates that the fault localization results become more credible. Even though there were possibly some faults, it would still provide valuable references. By comparing the black lines in Figure 8 with Figure 9, which represented the sum of the fault probabilities of correct fault location (deviation = 0) or adoptable deviation (deviation = 1), it can be seen that the localization accuracies of the proposed CNN-DIP method (93.5%) increased significantly than that of the traditional method (88%); moreover, with the increase of the noise, its accuracies decreased slowly, it has a powerful capacity to resist interference. The diagnostic results showed that the CNN-DIP method has higher reliability. 

## 4. Conclusions

In this paper, a novel fault identification (CNN-DIP) method combining deep learning algorithm and DIP methods was proposed. The overall fault identification accuracies without background noises raised from 61.7% to 91.67%. As the noises increased, the fault identification accuracies of the proposed CNN-DIP method decreased much slower than the traditional one, which was 74.1% when SNR = 0.2. In this case, the diagnostic accuracy based on traditional method was 11.9%. These results illustrated two aspects: First, the feature extraction method based on deep learning could make the FRA process much more effective and adaptive compared with traditional methods. On the other hand, the proposed CNN + DIP method could be of better anti-interference ability. Considering if the deviations between fault localization results with the corresponding actual fault locations = 1, it could be considered as acceptable. In this situation, the fault identification accuracies raised from 88% to 93.5% (without background noises), and 46.5% to 80% (while SNR = 0.5), which means the method proposed in this paper has better fault tolerance. The conclusions of this paper are as follows:

- The proposed fault identification method based on a lightweight Convolutional Neural Network (MobileNet-V2) and DIP could improve the diagnostic adaptabilities and accuracies.
- Through noise analysis, the anti-interference ability of the proposed CNN-DIP method was compared with that of the traditional method, which indicates that the proposed method has good stabilities under strong interference.
- The fault localization deviations were studied. The results shown in Figure 8 and Figure 9 indicated that the proposed method has better fault tolerance and could provide more valuable supports.

## Figures and Tables

**Table 1:** Calculated winding electrical parameters.

Ground Capacitance *C_g_*	Disc-to-disc Capacitance *Cs*	Self-Inductance *L_s_*	Mutual Inductance		Resistance *R*
			*M_i_*_(*i* + 1)_	*M_i_*_(*i* + 2)_	
30.05 pF	582.98 pF	0.101 mH	0.079 mH	0.053 mH	261 mΩ

**Table 2:** Monitoring data visualization method.

Type of Data	Example of Data Visualization Process	Samples
*Monitoring image*	No conversion required.	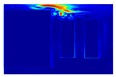
*Waveform image*	Can be trained directly.	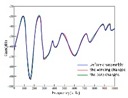
*Waveform data*	Decompose the data into characteristic spectrums; or the data can be directly drawn into a waveform diagram.	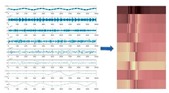
*Parameter value or text expression*	Draw a suitable image according to the features of the values; or convert through text visualization technology.	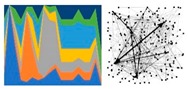

**Table 3:** Fault diagnosing dataset of transformers.

Labels	Dataset	Color Adjustment	Rotate	Crop	Noises	Sum
00	10	60	80	50	50	250
11/21/31	30	180	240	150	150	750
12/22/32	30	180	240	150	150	750
13/23/33	30	180	240	150	150	750
14/24/34	30	180	240	150	150	750
15/25/35	30	180	240	150	150	750
16/26/36	30	180	240	150	150	750
17/27/37	30	180	240	150	150	750
Total	220	1320	1760	1100	1100	5500

**Figure A1:**
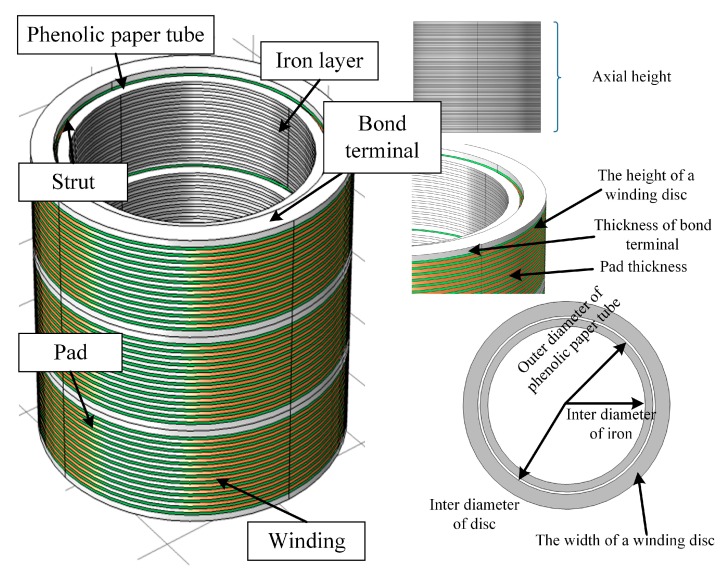
Labeling of transformer model parameters.

## Appendix A

The structural dimensions and insulation parameters of the diagnosing transformer in this paper are shown in Table A1.

**Table A1:** Dimensions of windings and the relative dielectric constants of insulation materials.

Structure	Size/mm	Structure	Size/mm	Material	Relative Dielectric Constant
Axial height	432	Pad thickness	10	Insulation paper	2.6
The height of a winding disc	6	The width of a winding disc	36.5	pad	4.5
Inter diameter of iron	196	Inter diameter of disc	221	Strut	4.4
Outer diameter of phenolic paper tube	215	Thickness of bond terminal	9	Bond terminal	4.4
Iron thickness*	0.1	Strut thickness*	4	Phenolic paper tube	3.8

For visual explanations, the sizes of the 3D model in Figure 2 were marked, as shown in Figure A1. Where the * represents that this parameter is simplified in 3D model.
